# Vertical Transmission of Group B *Streptococcus*, Prevalence, Associated Factors, and Antimicrobial Susceptibility Profile among Newborns Delivered at Health Facilities in Jigjiga City, Ethiopia

**DOI:** 10.1155/2024/5673366

**Published:** 2024-08-03

**Authors:** Addisu Tesfaye, Mahlet Fekede, Fasil Getu, Surafel Mekuria, Tigist Abebe, Daniel Tollosa, Shamil Barsenga, Bawlah Tahir, Abdurahman Kedir Roble, Sara Tesfaye, Muluken Walle

**Affiliations:** ^1^ Department of Medical Laboratory Science College of Medicine and Health Sciences Jigjiga University, Jigjiga, Ethiopia; ^2^ Department of Pediatrics College of Medicine and Health Sciences Jigjiga University, Jigjiga, Ethiopia; ^3^ Department of Midwifery School of Nursing College of Medicine and Health Science Jigjiga University, Jigjiga, Ethiopia

## Abstract

**Background:**

Group B *Streptococcus* (GBS) colonizes the rectovaginal area of women and vertically transmitted to neonates. This bacterium has been linked to severe neonatal complications including pneumonia, septicemia, and meningitis. GBS shows an increased resistance to commonly used antibiotics. Thus, we assessed the vertical transmission, contributing factors, and antimicrobial resistance patterns of GBS among newborns delivered at selected Hospitals in Jigjiga City.

**Methods:**

Hospital-based cross-sectional study was conducted from 1^st^ June 2022 to 30th April 2023. A total of 849 pregnant women admitted to delivery wards from two hospitals were screened for GBS colonization. Subsequently, 162 GBS-colonized pregnant women and their newborn babies were included. A semistructured questionnaire and a review of medical records were used to collect the sociodemographic and clinical characteristics of the study participants. Trained nurses collected swab samples from the vaginal-rectal area of pregnant women and the nasal, ear canal, and umbilical areas of newborn babies. Samples were inoculated on Todd Hewitt broth media supplemented with gentamycin and nalidixic acid and then subcultured on blood agar. Colony characteristics, Gram stain, and catalase test were used for identification. All gram-positive cocci, B-hemolytic, and catalase-negative isolates were further identified using Christie–Atkins–Munch–Petersen and a bacitracin test. The modified Kirby-Bauer disk diffusion method was used for antimicrobial susceptibility testing. Data were analyzed using SPSS version 26. Logistic regression analysis was used to determine the factors associated with vertical transmission of GBS, and statistical significance was set at *p* values <0.05.

**Result:**

The overall vertical transmission rate was 41.4% (67/162). History of preterm labor (Adjusted odds ratio (AOR) = 2.25; 95% CI: 1.11, 4.59), history of urinary tract infection (UTI) at current pregnancy (AOR = 2.25; 95% CI: 1.11, 4.59), and prolonged rupture of membranes greater than 18 hours (AOR = 2.23; 95% CI: 1.13, 4.4) were significantly associated with vertical transmission of GBS from previously colonized mothers to their newborn babies. Regarding GBS antibiotic susceptibility profile, a significant degree of resistance was observed to penicillin (29.9%), tetracycline (22.4%), ampicillin (20.9%), and clindamycin (19.4%).

**Conclusion:**

Our study documented a high prevalence of vertical transmission rate of GBS from pregnant women to their babies, with an overall transmission rate of 41.4%. The study identified the presence of antimicrobial-resistant GBS to penicillin, ampicillin, clindamycin, ciprofloxacin, and chloramphenicol. The organism was susceptible to vancomycin, followed by azithromycin, ceftriaxone, and erythromycin. Our study also reported MDR at 13.4%. Based on our findings, there is a need for antenatal culture-based GBS screening, maternal vaccination, and large-scale epidemiological and serotype identification studies to be put into practice in the study area.

## 1. Introduction

Group B *Streptococcus* (GBS), also known as *Streptococcus agalactiae*, is facultative anaerobic, encapsulated, beta-hemolytic, and Gram-positive bacteria [1]. It is catalase negative, bacitracin resistant, positive in the Christie–Atkins–Munch–Peterson (CAMP) test, and forms large white mucoid colonies on blood agar plates [2]. GBS colonizes the gastrointestinal and vaginal tracts of one third of pregnant women asymptomatically, as part of the normal flora [3]. Multiplication of these bacteria around the vaginal area can cause maternal morbidity during pregnancy [4]. Newborns from GBS-positive pregnant women are at substantial risk of infection [5]. About 50–75% of infants born to GBS-positive mothers become colonized at birth [6]. Microorganisms are transmitted vertically from the maternal vaginal or anorectally colonized mucosa throughout labor or in utero [7]. As a result, infants may develop colonization of their skin, oral cavity, nasopharynx, vagina, and various mucosal surfaces [8].

It has been reported that GBS is the principal cause of neonatal sepsis, meningitis, and pneumonia which are attributed to infant mortality [9]. Neonatal diseases caused by GBS are categorized as early-onset disease (EOD) and late-onset disease (LOD) [2]. The primary risk factor for EOD is rectovaginal colonization in pregnant women with GBS before or during delivery, which occurs in the first week of life (0–6 days) with pneumonia or respiratory distress, commonly advancing to sepsis [4].

Newborns with LOD often range in age from the 7th day to the 89th day. They typically appear with bacteremia and have a high meningitis complication rate. They arise from maternal, nosocomial, or community sources [10].

GBS is the leading cause of early invasive infection in newborns worldwide [11]. This bacterium has been associated with 205,000 EOD cases, 3.5 million preterm deliveries, 57,000 stillbirths, 90,000 infant deaths, and more than 10,000 new cases of neurodevelopmental abnormalities per year globally [11]. The fatality rate for infants with EOD disease is estimated to be 4–6% [12]. Moreover, surviving infants may experience long-term disabilities including hearing loss, vision loss, and mental retardation [13]. Most severe neonatal cases, stillbirths, and infant fatalities occur in Africa [14].

Different factors can increase the risk of neonatal GBS colonization and infection. The main factors include heavy maternal colonization, lack of intrapartum antibiotic prophylaxis (IAP), prolonged rupture of membranes, prematurity, previous newborns with EOD, lower antenatal care (ANC) visits, prelabor rupture of membranes, intrapartum maternal fever, history of preterm delivery, and history of urinary tract infection (UTI) during the current pregnancy [15–20].

Administration of IAP treatment with penicillin or amoxicillin to GBS-colonized pregnant women, pregnant women with risk factors, and pregnant women with unknown colonization status is considered the most effective approach for preventing EOD in newborns [8]. Penicillin is still considered the first-choice antibiotic for IAP to prevent EOD and to treat GBS disease [21]. However, resistance to penicillin and fluoroquinolones has recently been observed [22]. Over the last two decades, GBS has been increasingly resistant to a variety of antibiotics, including macrolides (such as erythromycin) and lincosamides (such as clindamycin), from <5% to common resistance of 20–30% [4, 23]. Antimicrobial resistance is difficult to treat and poses a serious health risk, causing a sharp increase in GBS infections in newborns. One issue prompting this concern is the increased prophylactic use of antibiotics, without appropriate bacterial culture and screening. Therefore, the antimicrobial resistance of GBS to different antibiotics is a major issue worldwide [24]. Furthermore, most developing countries like Ethiopia do not have clear guidelines for the prevention of vertical transmission of GBS from GBS-positive mother to neonates due to limited information regarding it. Thus, the current study aimed to investigate the vertical transmission rate, associated factors, and antimicrobial resistance patterns of GBS among newborns delivered at selected health facilities in Jigjiga city.

Prevention techniques, including universal antenatal GBS screening, identification of risk factors, and antibiotic prophylaxis at delivery, have been capable of significantly limiting the disease [15].

## 2. Methods

### 2.1. Study Design, Area, and Period

A hospital-based cross-sectional study was conducted from June 01, 2022, to April 30, 2023, at two selected Hospitals in Jigjiga city, the administrative city of the Somali regional state. The city is located 636 km southeast of Addis Ababa, Ethiopia's capital city. According to data from the Central Statistical Agency in 2015, the estimated total population of Jigjiga City is 304,000 [25]. At the time of data collection, three hospitals and four health centers offered delivery services in Jigjiga. The largest client loads for delivery services were used to determine the selection of two hospitals: Karamara General Hospital (KGH) and Jigjiga University Sheik Hassen Yabere Referral Hospital (JUSHYRH).

### 2.2. Population

All pregnant women admitted to the delivery rooms and their newborn babies at JUSHYRH and KGH were the source population of the study. The Study population consisted of newborn babies delivered from GBS-colonized pregnant women.

### 2.3. Eligibility Criteria

All GBS-colonized pregnant women admitted to the labor and delivery rooms at JUSHYRH and KGH during the data collection period and their newborn babies were included in this study. Pregnant women who used vaginal cream, lubricants, traditional sterilizers (vinegar), and antibiotics in the last two weeks before data collection were excluded from the study since the abovementioned factors will give false negative results. Participants who refused to provide informed consent were also excluded from this study.

### 2.4. Study Variables

The vertical transmission rate of GBS colonization and antimicrobial resistance patterns of GBS isolates were the dependent variables of the study, while sociodemographic characteristics and clinical/obstetrical features were the predictor variables. Sociodemographic characteristics included newborn sex, maternal age, residence, marital status, occupation, and educational status. Clinical/obstetrical variables included prolonged labor, maternal fever, history of stillbirth, history of contraceptive use, gravidity, history of neonatal death, gestational week, outcome of previous deliveries, prenatal care, GBS-contributing diseases, ANC visit, UTI, prolonged rupture of membranes >18 h, and preterm labor (<37 weeks). Moreover, variables of newborns, including the APGAR score (appearance, pulse, grimace, activity, and respiration), status of newborns born during delivery, sex of newborns, and newborn weight during delivery were studied.

### 2.5. Sample Size Determination and the Sampling Technique

The required sample size was calculated using a single-population proportion formula based on the proportion of vertical transmission of GBS at delivery from previous report in southern Ethiopia, i.e., 11.9% [23].

(1) $$\begin{array}{c} N={\left(Z_{\left(\alpha /2\right)}\right)}^{2}\left(\frac{p\left(1-p\right)}{d^{2}}\right)={\left(1.96\right)}^{2}\left(\frac{0.119\left(1-0.119\right)}{{\left(0.05\right)}^{2}}\right)=162\left(\text{GBS colonized mothers and their newborn babies}\right), \end{array}$$

where *N* = the desired sample size, *z* = standard normal distribution value at 95% CI, *d* the acceptable margin of error, and *p* the proportion of GBS vertical transmission from a previous study.

The calculated sample size was allocated proportionally to each selected hospital based on the estimated number of pregnant women visiting the delivery ward in the three data collection months: 720 in JUSHYRH and 530 in KGH. The number of samples in each selected hospital was determined by the proportional allocation method using the following formula:

(2) $$\begin{array}{c} n=\left(\frac{N1}{\text{Nt}}\right)\times \text{nt}, \end{array}$$

where *n* is the sample size, *N*1 is the average number of pregnant women attending the delivery ward in each hospital, Nt is the total number of pregnant women attending the delivery ward in both hospitals, and nt is the determined sample size.

Therefore, 162 GBS-colonized pregnant women (93 from JUSHYRH and 69 from KGH) admitted to the delivery ward and their newborn babies were included in the study. Participants were recruited from the study population using a convenient sampling technique.

### 2.6. Data Collection

A semistructured questionnaire with a review of medical records was used to collect the sociodemographic, obstetric, and clinical characteristics of the study participants to investigate the factors associated with vertical transmission of GBS. The questionnaire was developed based on the WHO and Center for Disease Control and Prevention (CDC) guidelines and referring related literatures [26, 27].

### 2.7. Specimen Collection and Bacteriological Procedures

Vaginal rectal swabs were collected from pregnant women admitted to the labor and delivery room by brushing the lower vagina and rectum with sterile cotton swabs by trained nurses following universal precautions [28–30]. Swabs were also collected from the nasal area, ear canal, and umbilical areas of newborns within 30 min of birth, following the standards of the American Society for Microbiology (ASM). Bacteriological analysis was performed following the methods described by the CDC, American College of Obstetrician's Gynecologists, and ASM [30, 31]. Samples were inoculated in Todd Hewitt broth media supplemented with gentamycin (8 *μ*g/ml) and nalidixic acid (15 *μ*g/ml) and incubated at 37°C with 5% CO_2_ for 24 h. Then, they were subcultured on blood agar plates and incubated at 37°C with 5% CO_2_ for 24 h–48 h. Colony characteristics, Gram staining, and catalase tests were used for presumptive identification. All Gram-positive cocci and B-hemolytic and catalase-negative isolates were further identified using CAMP and bacitracin tests. The CAMP test was used to differentiate CAMP-positive GBS from other beta-hemolytic *Streptococci* while bacitracin test was used to distinguish GBS from group A *streptococci*, which are both B-hemolytic [28, 32].

### 2.8. Antimicrobial Susceptibility Testing

The Kirby–Bauer disk diffusion method was used to perform the antimicrobial sensitivity test (AST). Around 4-5 GBS colonies with the same morphology were added to 5 ml of sterile physiological saline to create a bacterial suspension using McFarland's standard (0.5) as a reference. After the addition of 5 percent defibrinated sheep blood, the suspension was inoculated onto Mueller–Hinton agar plates. The antibiotic disks were positioned and incubated at 35–37°C with 5% CO_2_. The antibiotics suggested by the Clinical and Laboratory Standards Institute (CLSI) guidelines were selected and tested against GBS [33]: penicillin *G* (P, 10 *μ*g), ampicillin (AMP, 10 *μ*g), clindamycin (CLY, 2 *μ*g), erythromycin (E, 15 *μ*g), chloramphenicol (C, 30 *μ*g), ciprofloxacin (CIP, 5 *μ*g), ceftriaxone (CRO, 30 *μ*g), vancomycin (VA, 30 *μ*g), azithromycin (AZM, 15 *μ*g), and tetracycline (TE, 30 *μ*g). The zone of inhibition around the antibiotic disks was measured using a calibrated ruler and interpreted as sensitive, intermediate, or resistant using a standard chart [28, 33].

### 2.9. Data Analysis

The data were analyzed using SPSS V26. Frequency tables, graphs, and other statistical summary measures were used to summarize the results. Bivariate logistic regression analysis was used to assess the potential factors contributing to vertical transmission. Variables with *p* − value ≤ 0.25 were entered into the multivariate logistic regression analysis. Crude odds ratios (COR) and adjusted odd ratios (AOR) were used to determine the strength of the association. Statistical significance was set at *p* values  < 0.05.

### 2.10. Data Quality Control

The questionnaire was pretested and training was provided to the data collectors before data collection. Standard operating procedures (SOPs) were followed for sample collection, transportation, and bacteriological processing. Any physical changes (growth of microorganisms) and expiration dates were checked before using the reagents and culture media. The international control bacterial strains *Streptococcus agalactiae* (ATCC 27956), *Staphylococcus aureus* (ATCC 24923), *Streptococcus pyogenes* (ATCC 19615), and *Escherichia coli* (ATCC 25922) were used as quality controls for the culture and antimicrobial susceptibility test (AST) [34, 35].

### 2.11. Operational Definitions

- Vertical transmission: transmission of the pathogen from mother to neonate during delivery
- GBS colonization: a condition by which carrying GBS in/on bodies with growth and multiplication without showing tissue invasion or damage [35].
- Early-onset disease: diseases that appear from birth to 6th completed days [4].
- Late-onset disease: diseases that appear in infants between the 1st week and 89 days of age [4].
- MDR: a condition by which an organism is resistant to three and more class of antibiotics [33].

## 3. Results

### 3.1. Sociodemographic, Obstetric, and Clinical Characteristics of Study Participants

A total of 849 pregnant women admitted to the delivery ward from two hospitals were screened for GBS colonization; 478 pregnant women were from JUSHYRH, while the remaining 371 were from KGH. Of these, 162 GBS-colonized mothers (93 from JUSHYRH and 69 from KGH) and their newborns were recruited for the study. Majority of GBS-colonized mothers were married (67.3%), urban residents (72.8%), and aged greater than 25 years (75.3%). Moreover, most of the study participants cannot read and write (38.3%), followed by primary level of education (26.5%) and secondary level of education (21%) (Table 1).

The clinical characteristics of the study participants showed that majority of them were multigravida (128 (79%)) and above 36 weeks of gestation during delivery (146 (90.1%)). Most of the mothers had ANC visits during pregnancy (120 (74.1%)) and negative for HIV (155 (95.7%)). Furthermore, majority of pregnant women had prolonged rupture of membrane (64 (39.5%)), history of UTI during current pregnancy (63 (38.9)), history of neonatal death (43 (26.5%)), and history of preterm labor (53 (32.7%)). Of the newborns, majority of newborns were females (103 (63.6%)) and <2500 grams at birth (118 (72.8%)). Approximately 6.2% of newborns had a APGAR score of <7 at 5 min (Table 2).

### 3.2. Vertical Transmission and Associated Factors of *Group B Streptococcus* from Mothers to Newborns

**Figure 1:**
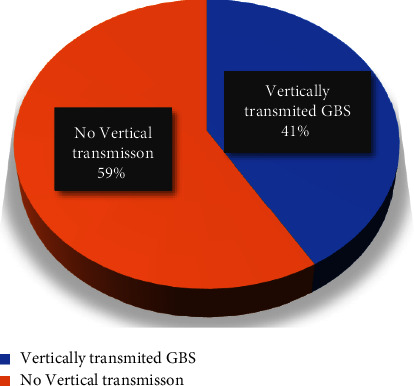
Proportion of GBS vertical transmission from mothers to newborns during delivery at JUSHYRH and KGH.

A total of 162 GBS-colonized pregnant women and their paired babies were included in this study. The overall vertical transmission rate was 67 (41.4%). All newborns colonized by this bacterium descended from previously colonized mothers (Figure 1).

The results of bivariable and multivariable logistic regression showed that a history of preterm labor (<37 weeks), history of UTI at current pregnancy, and prolonged rupture of membranes greater than 18 hours were significantly associated with vertical transmission of GBS from previously colonized mothers to their newborns. Mothers with a history of preterm labor (<37 weeks) were 2.25 times more likely to transmit GBS vertically to their newborns during delivery (AOR = 2.25; 95% CI: 1.11, 4.59). Whereas mothers who had a history of UTI at current pregnancy were 2.37 times more likely to transmit GBS vertically to their newborns during delivery (AOR = 2.37; 95% CI: 1.19, 4.71). In addition, mothers who had prolonged rupture of membrane greater than eighteen-hour were 2.23 times more likely to transmit GBS vertically to their newborns during delivery (AOR = 2.23; 95% CI: 1.13, 4.4) (Table 3).

### 3.3. Antimicrobial Susceptibility Profile of *Group B Streptococcus* Isolated from Newborns

**Figure 2:**
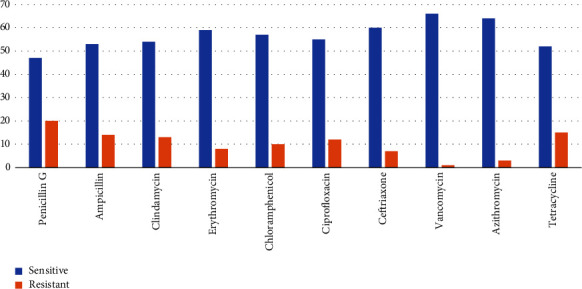
AST profile of GBS isolated from newborns at delivery at JUSHYRH and KGH.

We performed AST using ten AST disks for 67 GBS isolates that were vertically transmitted to the newborns. According to our study, 77.6% (52/67) of the isolates showed resistance to at least one antibiotic. Of the total drug drug-resistant isolates, 13.4% (9/67) were multidrug resistant (MDR) (Table 4). Out Of 67 GBS isolates, 20 (29.9%) were resistant to penicillin, followed by tetracycline 15 (22.4%), ampicillin 14 (20.9%), clindamycin 13 (19.4%), and ciprofloxacin 12 (17.9%). The organism was found to be susceptible to vancomycin (66 (98.5%)), followed by azithromycin (64 (95.5%)), ceftriaxone (60 (89.6%)), and erythromycin (59 (88.1%)) (Figure 2).

## 4. Discussion

Vertical transmission of GBS is a serious clinical and public health concern [36]. This bacterium is associated with EOD, preterm delivery, stillbirth, infant death, and neurodevelopmental abnormalities. The most severe neonatal cases, stillbirths, and infant fatalities occur in Africa [14, 37, 38].

In this study, the overall vertical transmission rate of GBS was 41.4% (95% CI: 33.7, 49.4%). This finding is in line with studies in Ethiopia which were conducted by Yadeta et al. [15] and Fantahun et al. [19], who reported a 45.02% and 47.6% rate of vertical transmission, respectively. However, this finding is higher compared to a study conducted by Le Doare et al. in Germany (11.2%) [9], Chen et al. in China (0.7%) [39], and Shah et al. in India (3.23%) [40]. Furthermore, the findings were higher compared to those in Africa, which were reported by Matee et al. in Kenya (8.9%) [41], Alemseged et al. in Ethiopia (11.9%) [42], and Ali et al. in Ethiopia (7.4%) [43]. A possible reason for this difference might be due to variations in study design, sample size, lifestyle, and prevalence of bacteria in the populations. On the other hand, the vertical transmission rate of GBS in this study was lower compared with studies conducted by Gizachew et al. [18] and Ali et al. [5] in Ethiopia, who reported 63.3% and 59.1% of vertical transmission rates, respectively. A possible reason for this difference might be variations in the study design, sample size, and lifestyle.

Antimicrobial resistance patterns were performed for GBS isolates from newborns by using different AST disks. Most drugs were used as the first-line treatment for the management of the case. Out of the total isolates, 77.6% resisted at least one drug test. Nine of the isolates (13.4%) were MDR. Of the total GBS isolates, approximately 29.9% were resistant to be resistance towards penicillin, followed by tetracycline (22.4%), ampicillin (20.9%), clindamycin (19.4%), ciprofloxacin (17.9%), chloramphenicol (16.4%), and erythromycin (14.9%). This could be due to the increasing trend of antimicrobial resistance to these antibiotics over time. This finding is in line with those of other studies conducted in Switzerland, which shows 14.5% and 8.2% resistance for erythromycin and clindamycin, respectively [44], for California and New York [45], Tehran, Iran, which shows 97.6%, 24.4%, and 14.6% resistance towards tetracycline, erythromycin, and clindamycin, respectively [46], Italy [47], in subregions of Africa [48], Cameron [49], Bahir Dar [36], Gondar [16], Southern Ethiopia [23], Southwest Ethiopia [17], and there is also one study in our study area and shows resistance profile for ceftriaxone, erythromycin, ciprofloxacin, clindamycin, and tetracycline at 17.2%, 20.7%, 27.6%, 27.6%, and 34.5%, respectively [28].

The organism was shown higher susceptible towards vancomycin (98.5%) followed, azithromycin (95.5%), ceftriaxone (89.6%), and erythromycin (88.1%). Therefore, these antibiotics could be used for the management of GBS colonization and the prevention of vertical transmission in the study area. This finding is supported by studies conducted worldwide by Chinato [50], Switzerland [44], California and New York [45], Iran [46], Italy [47], Cameron [49], Chinato [50], Bahir Dar [36], Gondar [16], Southern Ethiopia [23], and Jigjiga [28].

Our study reports a higher level of resistance of GBS for penicillin and tetracycline which was consistent with the studies done in high resistance in five subregions of Africa, Gondar, and Jigjiga [28, 48, 49].

This study also found multidrug resistance at 13.4%, which is comparable to studies done in Mekele (15.8%) and Jigjiga (13.79%) [28, 42].

This moderate antibiotic resistance found in our study area could be explained by a variety of factors, such as the excessive and improper use of antibiotics in the study area, which lacks regular antimicrobial susceptibility testing facilities and loose regulatory practices. The majority of the antimicrobials on this list can be bought and used without a prescription at community pharmacies. This would also contribute significantly to the high level of antibiotic resistance found in this study.

In this study, history of preterm labor (AOR = 2.25; 95% CI: 1.11, 4.59), history of UTI at current pregnancy (AOR = 2.25; 95% CI: 1.11, 4.59), and prolonged rupture of membranes greater than 18 h (AOR = 2.23; 95% CI: 1.13, 4.4) showed a statistically significant association with the vertical transmission of GBS. GBS generates extracellular membrane vesicles via virulence factors and toxins. This leads to extraplacental membrane thinning, collagen breakdown, and preterm birth [51]. The cytokeratin network in the amniotic epithelium becomes dysfunctional because of GBS infection of the choriodecidua, which weakens the membrane and leads to early membrane rupture. Membrane rupture increases the risk of infection because it leaves the fetus and amniotic fluid, which is a favorable environment for bacterial growth [52]. As GBS is one of the causative agents of UTI, mothers with UTI have a higher risk of vertical transmission of GBS [17]. This finding is supported by the studies conducted by Kim et al. in Korea [53], Dai et al. in China [54], Shah et al. in India [17], Yerumoh et al. in Nigeria, and several studies conducted in Ethiopia. There were also studies conducted in Ethiopia (Harar, Gondor, southwest Ethiopia, Northwest Ethiopia, Addis Ababa, and Adigrat), which are in line with our study and show that prelabor rupture of membranes at term, prolonged rupture of the membrane ≥18 hours, intrapartum maternal fever, history of preterm delivery and history of urinary tract infection (UTI) during the current pregnancy, were factors reported to have a significant association with GBS vertical transmission [15–20].

## 5. Conclusion and Recommendation

The current study revealed a high prevalence of vertical transmission rate of GBS from pregnant women to their babies, with an overall transmission rate of 41.4%. History of preterm labor (<37 weeks), history of UTI at current pregnancy, and prolonged rupture of membranes greater than 18 h were significantly associated with vertical transmission of GBS from previously colonized mothers to their newborns. Moreover, the study identified that GBS has increased antimicrobial resistance to penicillin and high susceptibility to vancomycin compared with other drugs. These findings suggest the need for antenatal culture-based GBS screening, maternal vaccination, risk factor-based interventions, and regular follow-up of drug resistance patterns to ensure proper treatment. Large-scale epidemiological studies with larger sample sizes are recommended.

### 5.1. Limitation of the Study

Serotyping and molecular tests were not performed to further characterize the identified GBS isolates. The findings may not be generalizable to the entire community as the study was conducted in health facilities.

## Figures and Tables

**Table 1:** Sociodemographic characteristic of study participants attending at JUSHYRH and KGH, Jigjiga, Ethiopia.

Variable	Categories	Total frequency *N* (%)	Vertical transmission result	
			Vertically transmitted *N* (%)	No vertical transition *N* (%)
Maternal age	<25 years	40 (24.70)	13 (32.50)	27 (67.50)
	≥25 years	122 (75.30)	54 (44.30)	68 (55.70)

Marital status	Single	14 (8.60)	8 (57.1)	6 (42.9)
	Married	109 (67.30)	42 (38.5)	67 (61.5)
	Divorced	23 (14.20)	9 (39.1)	14 (60.9)
	Widowed	16 (9.90)	8 (50)	8 (50)

Residence	Urban	118 (72.80)	44 (37.3)	74 (58.6)
	Rural	44 (27.20)	23 (52.3)	21 (47.7)

Occupation	Civil servant	13 (8.00)	4 (30.8)	9 (69.2)
	Student	17 (10.50)	8 (47.1)	9 (52.9)
	Housewife	57 (35.20)	14 (24.6)	43 (75.4)
	Self-employee	22 (13.60)	9 (40.9)	13 (59.1)
	Daily labor	15 (9.30)	9 (60)	6 (40)
	Farmer	8 (4.90)	4 (50)	4 (50)
	Merchant	30 (18.50)	19 (63.3)	11 (36.7)

Educational status	Cannot read and write	62 (38.30)	28 (45.2)	34 (54.8)
	Primary school	43 (26.50)	18 (41.9)	25 (58.1)
	Secondary school	34 (21.00)	11 (32.4)	23 (67.6)
	College/university	23 (14.20)	10 (43.5)	13 (56.5)

**Table 2:** Clinical characteristic of study participants attending JUSHYRH and KGH, Jigjiga, Ethiopia.

	Categories	Total frequency *N* (%)	Vertical transmission result	
			Vertically transmitted *N* (%)	No vertical transition *N* (%)
*Variables of mothers*				
Gravidity	Primigravida	34 (21.00)	10 (29.4)	24 (70.6)
	Multigravida	128 (79.00)	57 (44.5)	71 (55.5)

Gestational age at delivery (weeks)	<36 weeks	16 (9.90)	7 (43.8)	9 (56.2)
	≥36 weeks	146 (90.10)	60 (41.1)	86 (58.9)

History of abortion	Yes	25 (15.40)	12 (48)	13 (52)
	No	137 (84.60)	55 (40.1)	82 (59.9)

History of stillbirth	Yes	41 (25.30)	18 (43.9)	23 (56.1)
	No	121 (74.70)	49 (40.5)	72 (59.5)

ANC visit	Yes	120 (74.10)	55 (45.8)	65 (54.2)
	No	42 (25.90)	12 (28.6)	30 (71.4)

HIV status of the mother	Positive	7 (4.30)	3 (42.9)	4 (57.1)
	Negative	155 (95.70)	64 (41.3)	91 (58.7)

Prolonged rupture of membrane >18 hr	Yes	64 (39.50)	33 (51.6)	31 (48.4)
	No	98 (60.50)	34 (34.7)	64 (65.3)

History of UTI at current pregnancy	Yes	63 (38.90)	35 (55.6)	28 (44.4)
	No	99 (61.10)	32 (32.3)	67 (67.7)

History of neonatal death	Yes	43 (26.50)	19 (44.2)	24 (55.8)
	No	119 (73.50)	48 (40.3)	71 (59.7)

History of preterm labor (<37 weeks)	Yes	53 (32.70)	30 (56.6)	23 (43.4)
	No	109 (67.30)	37 (33.9)	72 (66.1)

Maternal fever during delivery	Yes	24 (16.70)	11 (45.8)	13 (54.2)
	No	138 (83.30)	56 (40.6)	82 (59.4)

*Variables of newborns*				
APGAR score at fifth minute	<7	10 (6.20)	5 (50)	5 (50)
	>7	152 (93.80)	62 (40.8)	90 (59.2)

Status of new born during delivery	Alive	159 (98.10)	66 (41.5)	93 (58.5)
	Dead	3 (1.90)	1 (33.3)	2 (66.3)

Sex of new born	Male	59 (36.40)	22 (37.3)	37 (62.7)
	Female	103 (63.60)	45 (43.7)	58 (56.3)

New born weight during delivery	<2.5 g	118 (72.8)	54 (45.8)	64 (54.8)
	≥2.5 g	44 (27.2)	13 (29.5)	31 (70.5)

**Table 3:** Bivariate and multivariate analysis of factors related with GBS vertical transmission among study participants attending JUSHYRH and KGH, Jigjiga, Ethiopia.

Factors	Categories	Vertical transmission		Corollary (95% CI)	*p* value	AOR (95% CI)	*p* value
		Yes	No				
Maternal age	<25	13	27	1			
	≥25	54	68	1.64 (0.77–3.49)	0.19		

Residence	Rural	23	21	1.84 (0.91–3.70)	0.08		
	Urban	44	74	1			

Educational status	Cannot read and write	28	34	0.93 (0.35–2.45)	0.89		
	Primary	18	25	1.06 (0.38–2.97)	0.89		
	Secondary	11	23	0.39 (0.53–4.80)	0.39		
	Above college	10	13	1			

Marital status	Single	8	6	1			
	Married	42	67	2.12 (0.68–6.52)	0.18		
	Divorced	9	14	2.07 (0.53–6.56)	0.28		
	Widowed	8	8	1.33 (0.31–5.64)	0.69		

Gravidity	Primigravida	10	24	1			
	Multigravida	57	71	1.92 (0.85–4.35)	0.11		

History of abortion	Yes	12	55	1.37 (0.58–3.23)	0.46		
	No	13	82	1			

History of stillbirth	Yes	18	23	1.15 (0.56–2.35)	0.70		
	No	49	72	1			

HIV status of the mother	Positive	3	4	1.06 (0.23–4.92)	0.93		
	Negative	64	91	1			

Prolonged rupture of membrane	Yes	33	31	2.00 (1.05–3.81)	0.03^*∗*^	2.23 (1.13–4.40)	0.02^*∗*^
	No	34	64	1		1	

History of UTI at current pregnancy	Yes	35	28	2.61 (1.36–5.02)	0.004^*∗*^	2.37 (1.19–4.71)	0.01^*∗*^
	No	32	67	1		1	

Maternal fever during pregnancy	Yes	11	13	1.23 (0.51–2.96)	0.63		
	No	56	82	1			

History of neonatal death	Yes	19	24	1.17 (0.57–2.36)	0.66		
	No	48	71	1			

History of preterm labor (<37 weeks)	Yes	30	23	2.53 (1.29–4.97)	0.007^*∗*^	2.25 (1.11–4.59)	0.02^*∗*^
	No	37	72	1		1	

*Variables of newborns*							
APGAR score	<7	5	5	1.45 (0.40–5.22)	0.56		
	>7	62	90	1			

Status of newborn	Alive	66	93	1.41 (0.12–15.9)	0.77		
	Dead	1	2	1			

Sex of newborn	Male	22	37	1			
	Female	45	58	1.30 (0.67–2.51)	0.42		

Newborn weight	<2.5	54	64	2.01 (0.95 4.22)	0.06		
	≥2.5	13	31	1			

“^∗^” want to indicate variables those are significantly associated.

**Table 4:** Antibiotic susceptibility profile of GBS isolated from newborns at delivery at JUSHYRH and KGH, Jigjiga, Ethiopia.

Antibiotic susceptibility profile	Antibiotic categories	Number of isolates (%)
No resistance		15 (22.5%)

R1	Penicillin	3 (4.5%)
	Erythromycin	2 (3%)
	Clindamycin	1 (1.5%)
	Ceftriaxone	2 (3%)
	Tetracycline	5 (7.5%)
	Chloramphenicol	2 (3%)
	Ciprofloxacin	3 (4.5%)
	Ampicillin	1 (1.5%)

R2	Penicillin, tetracycline	2 (3%)
	Clindamycin, chloramphenicol	2 (3%)
	Erythromycin, clindamycin	2 (3%)
	Clindamycin, tetracycline	2 (3%)
	Chloramphenicol, ciprofloxacin	2 (3%)
	Ciprofloxacin, ceftriaxone	1 (1.5%)
	Penicillin, ampicillin	5 (7.5%)
	Ciprofloxacin, tetracycline	1 (1.5%)
	Penicillin, ciprofloxacin,	1 (1.5%)
	Clindamycin, ceftriaxone	1 (1.5%)
	Ceftriaxone, tetracycline	1 (1.5%)

R3 and more	Erythromycin, clindamycin, ciprofloxacin^(^^*∗*^^)^	2 (3%)
	Penicillin, chloramphenicol, ceftriaxone^(^^*∗*^^)^	1 (1.5%)
	Ampicillin, penicillin, tetracycline	3 (4.5%)
	Ampicillin, penicillin, ceftriaxone	1 (1.5%)
	Penicillin, chloramphenicol, ceftriaxone^(^^*∗*^^)^	1 (1.5%)
	Ampicillin, penicillin, tetracycline, ciprofloxacin^(^^*∗*^^)^	2 (3%)
	Ampicillin, penicillin, tetracycline, vancomycin^(^^*∗*^^)^	1 (1.5%)
	Erythromycin, clindamycin, azithromycin^(^^*∗*^^)^	2 (3%)

^*∗*^R1: resistance to one antibiotic; R2: resistance to two antibiotics; R3: resistance to three antibiotics.

## Data Availability

The datasets used to support the findings of this study are available from the corresponding author upon reasonable request.

## Abbreviations

ANC: Antenatal care
AOR: Adjusted odds ratio
ASM: American society for microbiology
AST: Antimicrobial susceptibility test
CAMP: Christie–atkins–munch–petersen
CLSI: Clinical and Laboratory Standards Institute
COR: Crude odds ratios
CPS: Capsular polysaccharide
EOD: Early onset of disease
EONS: Early-onset neonatal sepsis
GBS: Group B *streptococci*
IAP: Intrapartum antibiotic prophylaxis
IRB: Institutional Review Board
JUSHYRH: Jigjiga University Sheik Hassan Yabare Referral Hospital
KGH: Karamara General Hospital
LOD: Late-onset diseases
PCR: Polymerase chain reaction
PROM: Preterm premature rupture of membranes
SOP: Standard operating procedure
UTI: Urinary tract infection.
